# Repurposing azole antifungals as rapid bactericidal, membrane-disruptive therapeutics against *Clostridioides difficile*

**DOI:** 10.1128/spectrum.02900-25

**Published:** 2026-05-18

**Authors:** Ahmed A. Abouelkhair, Nader S. Abutaleb, Brice J. Stolz, Mohamed N. Seleem

**Affiliations:** 1 Department of Biomedical Sciences and Pathobiology, Virginia-Maryland College of Veterinary Medicine, Virginia Polytechnic Institute and State University 1757, Blacksburg, Virginia, USA; 2 Center for One Health Research, Virginia Polytechnic Institute and State University 1757, Blacksburg, Virginia, USA; Barnard College, New York, New York, USA

**Keywords:** *Clostridioides difficile* infection, azole antifungals, microbiota, simulated fluids, *Caenorhabditis elegans*, cell membrane permeabilization, scanning electron microscopy

## Abstract

*Clostridioides difficile* is a leading cause of healthcare-associated infections and remains an urgent global public health concern. Current FDA-approved therapies for *C. difficile* infection (CDI) are limited and are frequently associated with treatment failure and high rates of disease recurrence. To address this unmet clinical need, we employed a drug repurposing strategy focused on FDA-approved compounds with established safety profiles and limited systemic absorption characteristics, which are desirable for localized gastrointestinal infections. Through this approach, we identified the antifungal azoles miconazole, econazole, and tioconazole as potent inhibitors of *C. difficile*. These compounds exhibited robust *in vitro* activity against a diverse panel of clinical *C. difficile* isolates, with MIC_50_ values of 1, 2, and 2 µg/mL, respectively. Time-kill analyses demonstrated that all tested azoles exerted rapid bactericidal activity within 2 h, exceeding the killing kinetics of the current standard-of-care agents vancomycin and fidaxomicin, likely attributable to their membrane-disruptive mechanism that compromises cytoplasmic membrane integrity and leads to rapid cellular collapse. Additionally, the azoles displayed prolonged post-antibiotic effects (>12 h). Furthermore, the azoles exhibited limited activity against two representative members of the commensal intestinal microbiota (*Bifidobacterium* and *Lactobacillus* spp.) and retained antibacterial efficacy under physiologically relevant conditions, including high inoculum, variable pH, and simulated gastrointestinal fluids. Mechanistic studies supported membrane targeting as the primary mode of action. Azole exposure significantly disrupted bacterial membrane integrity, and scanning electron microscopy revealed marked morphological damage, including membrane distortion and cellular collapse, consistent with structural membrane damage. Notably, cholesterol supplementation increased *C. difficile* tolerance to azoles and other membrane-disrupting agents, suggesting that host cholesterol levels or dietary factors may influence CDI outcomes. Finally, in a *Caenorhabditis elegans* CDI infection model, all tested azoles significantly improved host survival following lethal *C. difficile* challenge. Collectively, our findings highlight the potential of azole antifungals as promising platforms that merit further investigation and modification in the pursuit of safer, more effective therapeutics for CDI treatment.

**IMPORTANCE:**

*Clostridioides difficile* infection (CDI) is a serious and recurrent intestinal disease for which current treatment options remain limited and often disrupt the protective gut microbiota, increasing the risk of relapse. In this study, we show that the antifungal drugs miconazole, econazole, and tioconazole exhibit rapid and sustained activity against *C. difficile* under conditions that mimic the gastrointestinal environment. Unlike existing therapies, these agents display minimal activity against commensal gut bacteria and provide prolonged post-antibiotic effects. We also demonstrate that cholesterol, a common host-derived molecule, modulates bacterial susceptibility to these drugs, revealing a previously underappreciated link between host factors and therapeutic efficacy. Finally, these compounds confer significant protection in an *in vivo Caenorhabditis elegans* model of CDI, highlighting their potential as candidates for repurposing and further development.

## INTRODUCTION

*Clostridioides difficile* is the leading cause of antibiotic-associated diarrhea, both in the United States and worldwide (1–3). The Centers for Disease Control and Prevention (CDC) has classified *C. difficile* as an urgent public health threat in its Antibiotic Resistance Threats Reports (4). *C. difficile* infection (CDI) is a major burden to the healthcare system, which was responsible for approximately 500,000 cases per year and 30,000 annual deaths in the United States in the past decade (3, 5). Recent multinational surveillance data encompassing 59 studies across 24 countries indicate that CDI continues to impose a substantial global burden. The highest incidence was reported in hospital-onset healthcare facility settings, reaching 5.31 cases per 1,000 admissions and 5.00 cases per 10,000 patient-days, while long-term care facilities reported rates as high as 44.24 cases per 10,000 patient-days. Recurrence was particularly frequent in community-acquired CDI (16.22%), and mortality rates reached 8.32% at 30 days and up to 16.05% overall (6, 7). Furthermore, the recent CDC’s Emerging Infections Program (EIP) report indicated that there were 117.2 CDI cases per 100,000 individuals across all EIP surveillance sites, including both community-associated (CA) and healthcare-associated (HA) settings. Although HA CDI has historically been primary, CA CDI cases now represent a significant portion of the total burden, with more than half of cases (62.3 cases per 100,000 individuals) being community-onset (8). Collectively, these data underscore the persistent and substantial impact of CDI despite ongoing prevention efforts. Additionally, the yearly cost burden of CDI is thought to surpass $4.6 billion, taking into account both immediate medical costs and the long-term financial consequences of severe and recurrent infections (9). Clinically, *C. difficile* can be asymptomatic in the digestive tract of 2%–5% of the population, but it can also cause a range of symptoms, from diarrhea to severe colitis, toxic megacolon, sepsis, and leaky gut (10, 11).

Toxins are the principal virulence factors of *C. difficile* and are responsible for the clinical manifestations of CDI. The organism produces two major exotoxins, toxin A (TcdA) and toxin B (TcdB), which disrupt epithelial barrier integrity and trigger mucosal inflammation (12, 13). Certain hypervirulent strains additionally produce the binary toxin (CDT); although its role in pathogenesis remains debated, several studies have associated CDT-producing strains with increased disease severity and higher case fatality rates (14–17). The emergence of hypervirulent epidemic strains, such as the PCR ribotype 027 and North American pulso-type 1 strains, has further exacerbated disease severity and contributed to numerous outbreaks worldwide (18–20). In addition to toxin production, spore formation plays a central role in CDI transmission and recurrence. *C. difficile* spores can persist on inanimate surfaces for prolonged periods and exhibit marked tolerance to environmental stressors, including many disinfectants and antibiotic exposure, thereby facilitating environmental dissemination and reinfection (21, 22). Upon exposure to gut-derived bile acids, spores germinate into toxin-producing vegetative cells, which are the primary drivers of recurrent CDI (23).

Currently, vancomycin (VAN) and fidaxomicin (FDX) are the only FDA-approved antibiotics for treating CDI (24). Vancomycin is associated with a treatment failure rate of approximately 14% and a recurrence rate of 25%–30% (25, 26). While fidaxomicin has lower recurrence rates due to its limited disruption of the gut microbiota, its high cost limits accessibility in certain populations (27). Moreover, emerging resistance or reduced susceptibility to both agents has been reported (28, 29). Metronidazole, once widely used for CDI, is no longer recommended as a first-line therapy because of high treatment failure rates; it is now reserved for mild cases (30). The new Infectious Diseases Society of America and the Society for Healthcare Epidemiology of America (IDSA/SHEA) and European Society of Clinical Microbiology and Infectious Diseases (ESCMID) guidelines recommend metronidazole only if VAN and FDX are unavailable (31, 32).

In addition, tigecycline and rifaximin have demonstrated potential utility in the management of CDI, particularly as adjunctive therapies. Rifaximin has been employed as a “chaser” following vancomycin therapy to reduce recurrence, with multiple studies reporting beneficial effects on lowering CDI relapse rates (33, 34). However, rifaximin is not strongly endorsed by the ESCMID (32), whereas the IDSA/SHEA recommend its use in patients with multiple CDI recurrences (31). Furthermore, in cases of severe or refractory CDI, tigecycline has also been used off-label as an adjuvant treatment (35).

Fecal microbiota transplantation (FMT) is another effective approach for recurrent CDI, as it helps restore the gut microbial balance. Recently approved standardized FMT products, including Rebyota (Ferring Pharmaceuticals) and Vowst (Seres Therapeutics), have improved safety and consistency. However, FMT remains limited by high cost, concerns regarding pathogen transmission, particularly in immunocompromised patients, variability in donor material, and insufficient long-term safety data, and it is not approved for acute CDI treatment or for pediatric patients (36, 37). Despite an active antibiotic development pipeline, advancing new anti-*C*. *difficile* therapies has proven challenging. Several promising agents, including surotomycin and cadazolid, demonstrated strong preclinical efficacy but failed to meet noninferiority criteria compared to vancomycin in phase III clinical trials, leading to discontinuation of their development (38–40). Collectively, these shortcomings highlight the urgent need for novel, safe, and effective therapeutic strategies for CDI.

Traditional *de novo* drug discovery is a time-consuming and costly process, typically requiring 10–15 years and over $2 billion in investment (41). In contrast, drug repurposing offers a more efficient and cost-effective alternative, significantly reducing development timelines and expenses (42). Notably, several agents originally developed for other indications have been successfully repurposed for infectious diseases, including metronidazole, initially introduced as an antiprotozoal agent and later widely adopted for anaerobic bacterial infections (43), and niclosamide, an anthelmintic that has demonstrated antibacterial and antiviral activity in recent studies (44). Notably, ridinilazole, a compound initially developed for veterinary use, has emerged as a highly promising therapeutic for CDI in humans (45). These examples underscore the translational potential of repurposing strategies in accelerating therapeutic innovation. In a previous study, we employed a drug repurposing approach to screen the Pharmakon 1600 and Johns Hopkins compound libraries, identifying several azole antifungals with potent and selective activity against *C. difficile* (46). In the present study, we selected the most active azoles, miconazole, econazole, and tioconazole, for further investigation. We evaluated their antibacterial activity against a diverse collection of clinical *C. difficile* isolates, characterized their killing kinetics and post-antibiotic effects, and assessed the impact of bacterial inoculum size and environmental pH on efficacy. In addition, we examined compound stability and activity under simulated gastric and intestinal conditions, investigated their mechanisms of action, and evaluated *in vivo* efficacy using a *Caenorhabditis elegans* CDI model, which serves as a rapid, cost-effective whole-animal system to provide preliminary proof-of-concept for anti-*C*. *difficile* activity before advancing to mammalian models.

## MATERIALS AND METHODS

### Bacterial strains, chemicals, and media

*C. difficile* and gut microbiota isolates were obtained from the Biodefense and Emerging Infections Research Resources Repository (BEI Resources), the American Type Culture Collection (ATCC), and the CDC (Tables S1 and S2). Miconazole, tioconazole, L-cysteine, propidium iodide (Alfa Aesar), econazole (Acros Organics), fidaxomicin (Biosynth Carbosynth), nisin (Cayman Chemicals), and vancomycin hydrochloride (Gold Biotechnology) were purchased from commercial vendors. Brain heart infusion (BHI) broth, MRS broth, Anaerobic GasPak Sachets with Indicator (Becton Dickinson and Company), yeast extract, a BCA protein assay kit (Fisher Scientific), vitamin K, hemin, and cholesterol (Sigma-Aldrich), phosphate-buffered saline (PBS) (Corning), and a BacTiter-Glo kit (Promega) were acquired from commercial suppliers.

### Minimum inhibitory concentrations of azoles against *C. difficile*

The minimum inhibitory concentrations (MICs) for azole drugs were determined against *C. difficile* isolates via a 96-well microdilution assay as described in previous reports (47–49). Briefly, the drugs were added in triplicate, serially diluted in 96-well plates, and then incubated anaerobically with bacteria (~5 × 10^5^ CFU/mL) at 37°C for 48 h inside a GasPak anaerobic jar containing a GasPak EZ anaerobic sachet to generate anaerobic conditions. The plates were then examined for growth, and the MIC was determined as the lowest concentration of each drug that completely inhibited bacterial growth. The MIC_50_ and MIC_90_ were determined as the minimum concentrations of each agent that inhibited the growth of 50% and 90% of the tested isolates, respectively.

The minimum bactericidal concentration (MBC) of azoles was determined by plating 10 μL from wells showing no growth onto BHI-supplemented (BHIS) agar plates after MIC detection. The MBC was determined as the lowest concentration that reduced bacterial growth by 99.9% (50, 51).

### Killing kinetics of azole drugs against *C. difficile*

A time-kill assay was performed against *C. difficile* ATCC BAA-1870 and ATCC 43255 to evaluate the killing kinetics of the azole drugs as previously described (52, 53). Briefly, an overnight culture of *C. difficile* in the logarithmic growth phase was diluted in prereduced BHIS at ~10^6^ CFU/mL and exposed to 2× or 4× MICs of miconazole, econazole, and tioconazole. Vancomycin and fidaxomicin were included as positive controls, and dimethyl sulfoxide (DMSO) was run in parallel as a negative control. All cultures were incubated anaerobically at 37°C for 48 h. At predetermined time points (0, 2, 4, 8, 12, 24, and 48 h), 100 μL aliquots were removed from each culture, serially 10-fold diluted in pre-reduced BHIS broth, and plated onto pre-reduced BHIS agar plates. Plates were incubated anaerobically at 37°C for 24–48 h prior to colony enumeration. Bacterial counts were expressed as log_10_ CFU/mL. The lower limit of detection was 2 log_10_ CFU/mL. Bactericidal activity was defined as a ≥3 log_10_ CFU/mL reduction from the initial inoculum.

### Post-antibiotic effect

The post-antibiotic effects (PAEs) of the azoles, miconazole, econazole, and tioconazole were tested against *C. difficile* ATCC BAA-1870 and ATCC 43255 following previously described procedures (54). Briefly, a logarithmic phase culture of *C. difficile* was diluted in pre-reduced BHIS to ~10^6^ CFU/mL. Drugs (at concentrations equivalent to 5× MIC in triplicate) were incubated with bacteria anaerobically at 37°C for 1 h. DMSO was included as a control. After 1 h, each tube was diluted 1:300 with fresh prereduced BHIS broth. The tubes were then incubated anaerobically as described above. Samples were collected every 2 h, serially diluted, and plated onto pre-reduced BHIS agar plates. The PAE was calculated as follows: PAE  =  *T* − *C*, where *T* and *C* are the times required for the bacterial culture treated with drugs and the negative control (DMSO), respectively, to increase by 1 log_10_ CFU/mL.

### The impact of inoculum size on the anti-*C*. *difficile* activity of azoles

Broth microdilution assay (48, 55, 56) was utilized to determine the impact of *C. difficile* inoculum size on the activity of miconazole, econazole, and tioconazole against three clinical *C. difficile* isolates (ATCC 630, ATCC BAA-1870, and ATCC 43255). Three inocula were prepared: a standard inoculum (SI: ~5 × 10^5^ CFU/mL) and high inocula (HI: ~5 × 10^6^ and ~5 × 10^7^ CFU/mL) of each strain in BHIS broth, and these were tested against azole drugs and control antibiotics. The plates were then incubated as described above, and the MICs were determined.

### Evaluation of the antibacterial activity of azoles against representative strains of human gut microflora

The MICs of azoles were determined against representative strains of the normal human gut flora as previously described (57). A bacterial suspension equivalent to 0.5 McFarland was prepared and diluted in BHIS broth (for *Bifidobacterium*) and MRS broth (for *Lactobacillus*) to achieve a bacterial concentration of ~5 × 10^5^ CFU/mL. Serial dilutions of the test agents were incubated with gut bacteria before the MICs were determined. Anaerobic incubation was used for *Bifidobacterium* species, while 5% CO_2_ was used for *Lactobacillus*.

### The effect of pH on the anti-*C*. *difficile* activity of azoles

The broth microdilution method described above was used to assess the effect of pH on the anti-*C*. *difficile* activity of miconazole, econazole, and tioconazole against three *C. difficile* strains (ATCC 630, ATCC BAA-1870, and ATCC 43255). The drugs were tested throughout a pH range of 5–9 by adding either 1 M HCl or 1 M NaOH to the BHIS broth. The plates were then incubated as described above, and the MICs were determined.

### MIC determination of azole antifungals in simulated gastric fluid and simulated intestinal fluid

Simulated gastric fluid (SGF) and simulated intestinal fluid (SIF) were prepared as described previously (58, 59), and the pH of SGF was adjusted to 1.2 (gastric juice pH), whereas the pH of SIF was modified to 6.8 (intestinal pH).

The broth microdilution assay was utilized to determine the MICs of azole drugs and control antibiotics against *C. difficile* strains ATCC BAA-1870 and ATCC 630 after exposure to SGF and SIF for the corresponding periods.

### Propidium iodide uptake assay

The propidium iodide (PI) fluorescence uptake assay was carried out as described in previous reports (60, 61). A log-phase sample of *C. difficile* ATCC 43255 in BHIS broth was centrifuged and washed twice with prereduced PBS (OD_600_ ~ 1). Bacteria were subjected to miconazole, econazole, and tioconazole (2× and 5× MIC) and incubated anaerobically at 37°C for 3 h. The tubes were centrifuged at 6,000 rpm for 10 min, and each cell pellet was resuspended in a 1 mL working solution containing 5 μM PI. After 10 min of anaerobic incubation at 37°C, the samples were centrifuged and washed to remove the excess dye. The PI fluorescence in the cells was measured via a Biotek Synergy H1 Hybrid plate reader with excitation and emission wavelengths of 544 and 620 nm, respectively.

### Protein leakage assay

Protein leakage was investigated via a Bradford protein assay, as described previously (61, 62). A log-phase sample of *C. difficile* ATCC 43255 in BHIS broth was centrifuged and washed twice in prereduced PBS (OD_600_ ~ 1.2). The bacteria were treated with miconazole, econazole, or tioconazole in PBS cultures at two different concentrations (2× and 5× MIC each, in triplicate) and incubated anaerobically at 37°C for 3 h. The samples were subsequently centrifuged at 6,000 rpm for 10 min to separate the supernatants. In a 96-well plate, the supernatant of each sample and the Bradford working solution were combined at a 1:8 ratio. After a 30-min incubation in the dark, the OD_562_ was measured. The appropriate dilution of each treatment supernatant was prepared and used as a blank. The concentration of protein removed was calculated from the absorbance readings and the linear regression line of the standard curve using bovine serum albumin as the reference protein, which had a known concentration of ~750 µg/mL.

### ATP leakage assay

The amount of ATP leakage from *C. difficile* treated with azoles was determined as previously reported (48, 61, 63). A log-phase culture of *C. difficile* ATCC 43255 was centrifuged and washed twice with prereduced BHIS (OD_600_ ~ 1). Bacteria were exposed to miconazole, econazole, and tioconazole (2×, 5×, and 10× MIC) and incubated anaerobically at 37°C for 3 h. The tubes were then centrifuged at 10,000 × *g* for 10 min. The supernatants were collected, and the cell pellets were resuspended in prereduced BHIS. The ATP in the resuspended pellets (intracellular) and supernatants (extracellular) was quantified via a BacTiter-Glo Microbial Cell Viability Kit (Promega). Luminescence was detected via a Biotek Synergy H1 Hybrid plate reader (gain 135).

### Scanning electron microscopy

Scanning electron microscopy (SEM) was performed as previously reported (64, 65). *C. difficile* ATCC 43255 was cultured in BHIS broth to the mid-logarithmic phase (OD_600_ = 0.5–0.6). Azoles drugs were added at 5× MIC, while an equivalent volume of DMSO served as the negative control. Following a 3-h incubation, cells were harvested by centrifugation at 4,500 rpm for 5 min. The resulting pellets were washed three times with PBS and fixed in 2.5% glutaraldehyde prepared in PBS. Samples were stored at 4°C until further processing.

Fixed bacterial suspensions were filtered through 13 mm, 0.2 µm pore-size Nuclepore filters and washed with 0.1 M sodium cacodylate buffer for 15 min. Samples were then subjected to graded ethanol dehydration (15%, 30%, 50%, 70%, 95%, and 100% ethanol), with each step performed for 15 min. After dehydration, samples were dried using a critical point dryer. The dried filters were sputter-coated with platinum–palladium (Pd–Pt) to a thickness of 15 nm using a Leica EM ACE600 and subsequently examined with a JEOL IT500 scanning electron microscope.

### Effect of cholesterol supplementation on the membrane-disrupting activity of azole drugs

To further confirm the role of membrane disruption in the azole-mediated killing of *C. difficile*, a cholesterol competition assay was performed via a time-kill assay as described previously (66–68). Briefly, *C. difficile* cultures in the logarithmic growth phase were diluted in prereduced BHIS broth to achieve an inoculum of ~10^6^ CFU/mL and exposed to 2× MIC concentrations of the azole drugs, alongside vancomycin and fidaxomicin as controls. Cholesterol (Sigma) was freshly prepared in warm acetone and added to the cultures at final concentrations of 50, 100, 300, 500, and 700 μg/mL. Cultures were incubated anaerobically at 37°C, and aliquots were sampled at 0, 6, 12, and 24 h, serially diluted, and plated on prereduced BHIS agar to determine the bacterial CFU/mL.

### *In vivo* efficacy of azoles in a *C. elegans* CDI model

To examine the *in vivo* efficacy of azole drugs for combating CDI, we used a *C. elegans* infection model (69–71). The *C. elegans* model was selected as an established whole-animal screening platform that enables rapid, cost-effective, and ethically responsible evaluation of antimicrobial efficacy and host survival prior to validation in mammalian systems (72, 73). This model allows assessment of compound toxicity and therapeutic benefit in an intact organism while permitting higher-throughput testing than murine CDI models. The study was designed as a proof-of-concept *in vivo* evaluation to validate the promising *in vitro* activity of the tested azoles before progressing to more resource-intensive mammalian studies. *C. elegans* AU37 was grown on nematode growth medium at 15°C and fed *Escherichia coli* OP50. *C. difficile* ATCC 43255 (Ribotype-087) was cultivated overnight at 37°C in BHIS broth under anaerobic conditions, centrifuged, and washed twice with prereduced BHIS. The L4/young adult hermaphrodites were infected with 2 × 10^9^ CFU/mL *C*. *difficile* ATCC 43255 and incubated at 25°C for 3 h. After infection, *C. elegans* nematodes were aliquoted into microcentrifuge tubes (~20 worms per tube) and washed five times with M9 buffer. The infected worms were subsequently treated with azoles (at 1× MIC) and subsequently incubated for 14 days at 25°C. Vancomycin (at 1×, 5×, and 10× MIC) or PBS was used as a control. Worms were microscopically examined for survival throughout the experiment.

### Statistical analysis

Statistical analysis was conducted via GraphPad Prism version 10 for Windows (GraphPad Software, La Jolla, CA, USA). All experiments were performed in at least duplicate, each containing the biological replicates, and the data were plotted as mean ± standard deviation.

## RESULTS

### MIC determination of azoles

We assessed the anti-*C*. *difficile* activity of miconazole, econazole, and tioconazole (chemical structures shown in Fig. S1A, B, and C) against a panel of 33 *C. difficile* clinical isolates. As shown in Table 1, the azoles inhibited the growth of the tested strains at concentrations ranging from 1 to 4 μg/mL. Miconazole, econazole, and tioconazole inhibited 50% of the tested isolates (MIC_50_) at concentrations of 1, 2, and 2 μg/mL, respectively, while their MIC_90_ were 2, 4, and 4 μg/mL, respectively. Interestingly, the MICs of miconazole were comparable to those of the drug of choice, vancomycin, which inhibited 50% and 90% of the strains tested at concentrations of 0.5 and 1 μg/mL, respectively. Additionally, the MIC_50_ and MIC_90_ of fidaxomicin against the tested strains were 0.015 and 0.06 μg/mL, respectively (Fig. S1D).

**TABLE 1:** MIC and MBC values (μg/mL) of miconazole, econazole, and tioconazole against a panel of *C. difficile* clinical isolates^*c*^^,^^*d*^

*C. difficile* strain ATCC/BEI designation	Azole antifungal						Control antibiotic			
	Miconazole		Econazole		Tioconazole		VAN^*a*^		FDX^*b*^	
	MIC	MBC	MIC	MBC	MIC	MBC	MIC	MBC	MIC	MBC
NR- 49302	2	2	4	4	2	2	0.5	0.5	0.06	0.06
NR- 49304	2	2	2	2	4	4	0.5	1	0.06	0.06
NR- 49306	1	2	2	2	2	2	0.5	0.5	0.0078	0.0078
NR- 49307	2	2	2	2	4	4	0.5	0.5	≤0.0078	0.015
NR- 49308	1	1	2	2	2	2	0.25	0.5	0.015	0.015
NR- 49310	4	4	4	4	8	8	0.5	0.5	0.015	0.015
NR- 49313	2	2	2	2	4	4	1	1	0.03	0.06
NR- 49314	2	2	2	2	4	4	0.5	0.5	0.06	0.06
NR- 49319	2	2	2	2	4	4	0.5	0.5	0.03	0.03
NR- 49318	2	2	4	4	4	4	0.5	0.5	0.06	0.125
CDC-1067	1	1	2	2	2	2	2	2	0.06	0.06
CDC-1072	1	1	1	2	2	2	2	2	0.06	0.06
CDC-1076	1	1	1	1	2	2	2	4	0.03	0.03
CDC-1077	4	4	4	4	4	4	0.5	0.5	≤0.0078	0.0078
CDC-1078	1	1	2	2	4	4	1	2	0.03	0.03
CDC-1079	1	1	2	2	2	2	0.5	0.5	≤0.0078	0.015
CDC-1082	1	1	2	2	2	2	0.5	0.5	0.015	0.015
CDC-1083	4	4	4	4	2	2	1	1	0.03	0.03
CDC-1085	1	1	1	1	1	1	0.5	0.5	≤0.0078	0.015
CDC-1086	1	1	2	2	2	2	0.25	0.5	≤0.0078	0.0078
CDC-1087	1	1	1	1	2	2	0.5	0.5	≤0.0078	0.0078
CDC-1088	1	1	2	2	2	4	0.5	0.5	0.015	0.015
CDC-1089	2	2	2	2	4	4	0.25	0.5	0.06	0.06
CDC-1090	1	1	2	2	2	2	0.5	0.5	0.03	0.03
CDC-1092	2	2	2	2	4	4	2	2	0.015	0.015
CDC-1093	1	1	1	1	2	2	0.25	0.25	0.015	0.03
CDC-1094	2	2	4	4	4	4	0.5	0.5	0.03	0.03
CDC-1095	1	1	2	2	4	4	2	2	0.03	0.03
CDC-1096	1	1	2	4	2	2	0.25	0.25	0.015	0.015
ATCC 630	1	1	1	1	2	2	1	1	0.03	0.06
ATCC 43255	1	1	2	2	1	1	0.5	0.5	0.015	0.015
ATCC BAA-1870	1	1	1	1	1	1	1	2	≤0.0078	0.015
ATCC 9689	2	2	1	1	2	2	2	2	0.015	0.015
MIC_50_/MIC_90_	**1/2**		**2/4**		**2/4**		**0.5/1**		**0.015/0.06**	

^*a*^ VAN, vancomycin.

^*b*^ FDX, fidaxomicin.

^*c*^ MIC_50_ and MIC_90_ values were calculated from a total of 33 clinical *C. difficile* isolates tested in this study. MIC_50_ and MIC_90_ are defined as the minimum inhibitory concentrations that inhibit the growth of 50% and 90% of the isolates, respectively. NR strains were obtained from the Biodefense and Emerging Infections Research Resources Repository (BEI Resources), CDC strains were obtained from the Centers for Disease Control and Prevention, and ATCC strains were obtained from American Type Culture Collection.

^*d*^ Values shown in bold correspond to the cumulative MIC5050 and MIC90 values of three azoles against the tested isolates.

Notably, the minimum bactericidal concentration values for the azoles were either equal to or within one dilution of their corresponding MIC values against the tested isolates, indicating bactericidal activity against *C. difficile*. A similar pattern was observed for vancomycin and fidaxomicin (Table 1).

### Killing kinetics of azole drugs against *C. difficile*

**Fig 1:**
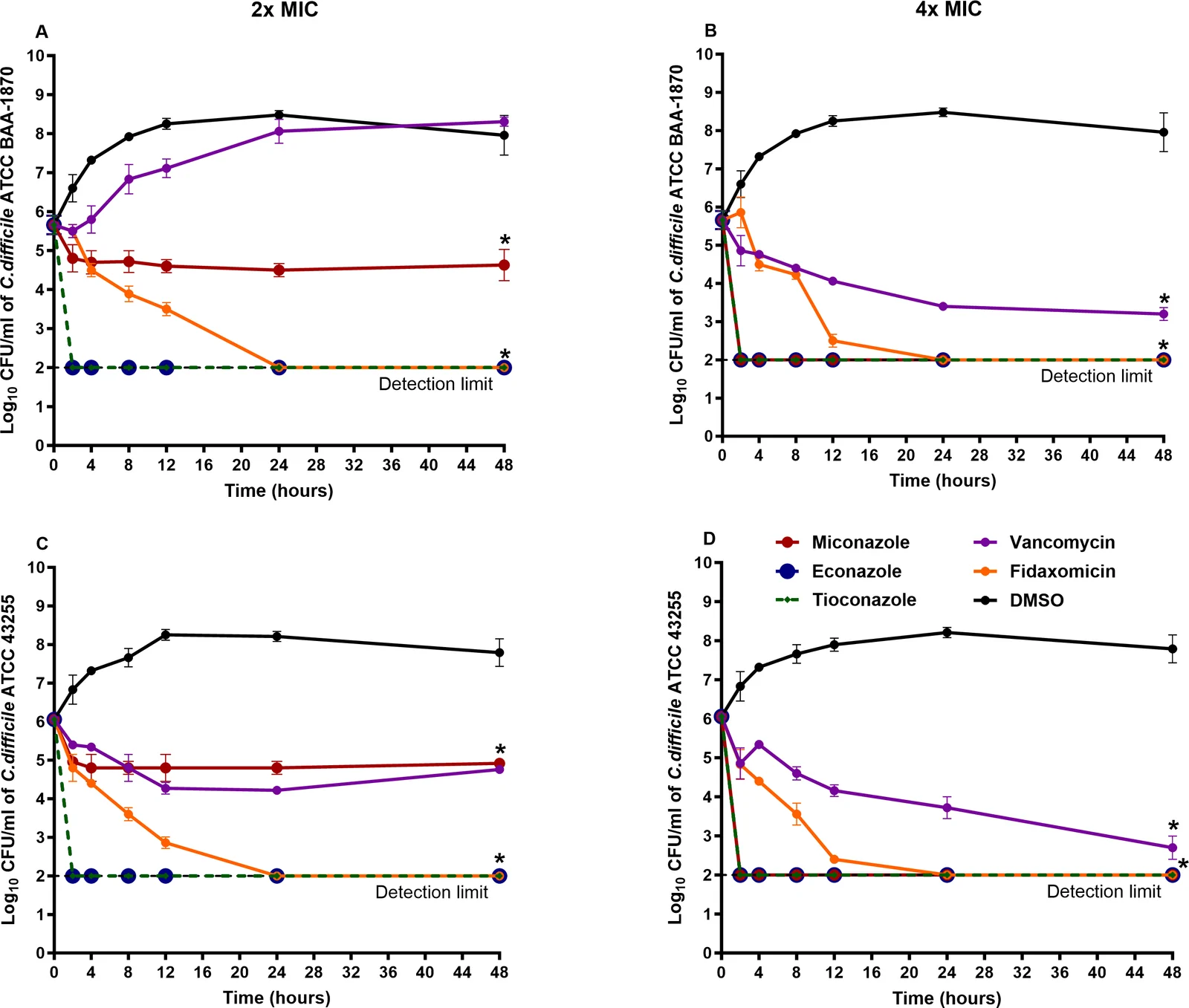
Time-kill kinetics of miconazole, econazole, and tioconazole against *C. difficile* ATCC BAA-1870 at 2× MIC (**A**) and 4× MIC (**B**) and *C. difficile* ATCC 43255 at 2× MIC (**C**) and 4× MIC (**D**). DMSO (solvent) served as a negative control. The error bars represent standard deviation values for each test agent. The data were analyzed via two-way ANOVA with *post hoc* Dunnett’s test for multiple comparisons. An asterisk indicates a statistically significant difference (*P* < 0.0001) between treatment with the test agent and the negative control (DMSO).

To confirm the bactericidal activity of azoles against *C. difficile*, we examined how rapidly they can reduce the burden of a high inoculum of *C. difficile* via a time-kill assay. As shown in Fig. 1, miconazole, econazole, and tioconazole exhibited rapid bactericidal activity against *C. difficile,* completely eradicating the high bacterial burden (below the detection limit) in only 2 h. In contrast, vancomycin caused a slow reduction in the *C. difficile* count, reducing the bacterial burden by only ~2 log_10_ CFU/mL after 24 h, whereas fidaxomicin was used for 24 h to reduce the bacterial burden below the limit of detection. Surprisingly, the azole drugs killed the high *C. difficile* burden much more quickly than both vancomycin and fidaxomicin did.

### Post-antibiotic effect of azole drugs

Following confirmation of the bactericidal activity of miconazole, econazole, and tioconazole against *C. difficile*, we evaluated their post-antibiotic effects to determine whether brief drug exposure could result in sustained bacterial suppression. All tested azoles exhibited prolonged PAE values against *C. difficile* strains ATCC BAA-1870 and ATCC 43255, ranging from 6 to ≥12 h and 8 to ≥12 h, respectively (Table 2). Econazole and tioconazole resulted in the greatest increase in PAE (≥12 h), followed by miconazole (6–8 h). Notably, the PAE values for the azoles exceeded those observed for the standard-of-care antibiotics vancomycin and fidaxomicin, which presented PAE values ranging from 2 to 6 h.

**TABLE 2:** Post-antibiotic effects of azole drugs against *C. difficile*

Drug	PAE (h)	
	*C. difficile* ATCC BAA-1870	*C. difficile* ATCC 43255
Miconazole	6	8
Econazole	≥12	≥12
Tioconazole	≥12	≥12
Vancomycin	2	4
Fidaxomicin	6	6

### The impact of inoculum size on the anti-*C*. *difficile* activity of azoles

The bacterial inoculum size is a critical factor influencing the efficacy of anti-*C*. *difficile* agents. To evaluate this, we assessed the impact of high inoculum levels (~5 × 10^6^ and ~5 × 10^7^ CFU/mL) on the activity of miconazole, econazole, and tioconazole compared with that of the standard inoculum (~5 × 10^5^ CFU/mL). Compared with the standard inoculum, the azoles retained potent activity, with MIC values ranging from 1 to 2 μg/mL. As the inoculum increased from 10^5^ to 10^6^ and 10^7^ CFU/mL, MIC values remained unchanged or increased by no more than one dilution (Table 3). A similar trend was observed with fidaxomicin. In contrast, vancomycin had a notable inoculum effect, with a fourfold increase in MIC values at the highest inoculum tested across the four *C. difficile* strains.

**TABLE 3:** MIC values (μg/mL) of azoles and control anti-*C*. *difficile* drugs against *C. difficile* clinical isolates at standard and high inoculum sizes^*a*^

Azole/control drug	MIC (μg/mL)								
	*C. difficile* ATCC 630			*C. difficile* ATCC BAA-1870			*C. difficile* ATCC 43255		
	10^5^	10^6^	10^7^	10^5^	10^6^	10^7^	10^5^	10^6^	10^7^
Miconazole	1	2	2	1	2	2	1	1	1
Econazole	1	2	2	1	2	2	2	2	2
Tioconazole	2	4	4	1	2	2	1	2	2
Vancomycin	1	1	4	1	1	4	0.5	0.5	2
Fidaxomicin	0.015	0.015	0.03	≤0.0078	≤0.0078	≤0.0078	0.015	0.015	0.015

^*a*^ 10^5^, standard inoculum (∼5 × 10^5^ CFU/mL); 10^6 ^and 10^7^, high inoculum sizes (∼5 × 10^6^ and ∼5 × 10^7^/mL).

### Evaluation of the antibacterial activity of azole drugs against representative strains of the human gut microflora

Alteration of the composition of the normal intestinal microbiome via the administration of antibiotics is a major predisposing factor for CDI (74, 75). Therefore, it is important to evaluate the anti-commercial activity of new anti*-C*. *difficile* agents. We tested the activity of the azoles against representative members of the human intestinal microflora, including *Bifidobacterium* and *Lactobacillus* (Table 4). Notably, miconazole, econazole, and tioconazole demonstrated limited activity against the *Bifidobacterium* strains tested, with MIC values ranging from 8 to 16 μg/mL. On the other hand, azoles did not show activity against *Lactobacillus* species, with MIC values of ≥64 μg/mL. In contrast, vancomycin and fidaxomicin inhibited the growth of the *Bifidobacterium* strains tested at concentrations of ≤2 µg/mL. At concentrations as high as 64 μg/mL, fidaxomicin inhibited the growth of the tested *Lactobacillus* strains, whereas vancomycin had either a negligible effect or no effect.

**TABLE 4:** MIC values (μg/mL) of azole and control anticlostridial drugs against normal human intestinal microbiota

Common gut bacteria	MIC (mg/mL)				
	Miconazole	Econazole	Tioconazole	VAN	FDX
*Bifidobacterium longum* HM-846	16	16	16	≤2	≤2
*Bifidobacterium angulatum* HM-1189	8	16	16	≤2	≤2
*Bifidobacterium longum* HM-845	16	16	16	≤2	≤2
*Bifidobacterium longum* HM-847	8	16	16	≤2	≤2
*Bifidobacterium breve* ATCC 15700	16	16	16	≤2	≤2
*Bifidobacterium breve* HM-856	8	16	16	≤2	≤2
*Bifidobacterium adolescentis* HM-633	4	8	8	≤2	≤2
*Lacticaseibacillus rhamnosus* ATCC 53103	>64	>64	>64	>64	4
*Lactobacillus gasseri* ATCC 19992	64	64	64	≤2	≤2
*Levilactobacillus brevis* ATCC 14869	64	64	64	>64	64
*Lactobacillus iners* HM-228	>64	>64	>64	>64	8
*Lactobacillus casei* ATCC 334	>64	>64	>64	>64	4

### The effect of pH on the anti-*C*. *difficile* activity of azoles

**Fig 2:**
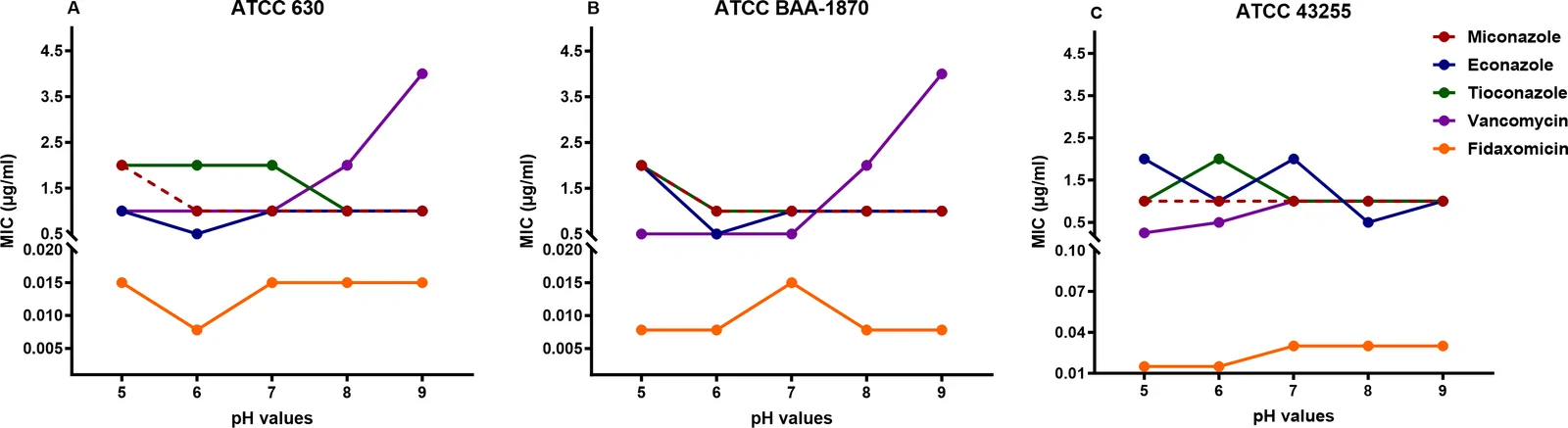
MICs (μg/mL) of azoles and control drugs against *C. difficile* ATCC 630 (**A**), *C. difficile* ATCC BAA-1870 (**B**), and *C. difficile* ATCC 43255 (**C**) at different pH values (6, 7, 8, and 9). The data are presented as MICs against the tested strains.

The colon, the primary site of *C. difficile* infection, maintains a pH range between 6.1 and 7.5 (76). To assess whether pH variations affect the anti-*C*. *difficile* activity of azoles, MICs were determined for three *C. difficile* isolates under acidic (pH 5–6), neutral (pH 7), and alkaline (pH 8–9) conditions. Miconazole maintained consistent activity (MIC = 1 µg/mL) across all tested pH values. Similarly, the MICs of econazole and tioconazole were either unchanged or increased by no more than one dilution under acidic or basic conditions (Fig. 2). In contrast, fidaxomicin and vancomycin exhibited decreased potency with increasing pH. For fidaxomicin, the MICs increased onefold under alkaline conditions (pH 8–9) against the *C. difficile* ATCC 43255 and ATCC 630 strains. The vancomycin MICs showed a more pronounced increase, increasing two- to fourfold as the pH increased.

### MIC determination of azole antifungals in simulated gastric fluid and simulated intestinal fluid

Assessing the stability of drugs, especially those to be administered orally, in the harsh conditions of the gastrointestinal tract (GIT) is important for identifying new anti-*C*. *difficile* agents. Therefore, we investigated the effects of SGF and SIF on the anticlostridial activity of all the potent azoles, including miconazole, econazole, and tioconazole (Tables 5 and 6). Each drug was incubated with either SGF or SIF, and the MICs were determined against two clinical strains of *C. difficile* at 0, 4, and 24 h. As shown in Table 5, miconazole retained its full activity after SGF exposure, with no change in MIC values over 24 h. Econazole and tioconazole exhibited MICs that were either unchanged or increased by no more than one dilution following incubation with SGF. Similarly, vancomycin and fidaxomicin showed stable activity after exposure to SGF, with MICs equivalent to or onefold higher than the control values. A comparable trend was observed after exposure to SIF, with all azoles, as well as vancomycin and fidaxomicin, maintaining their activity with MICs either unchanged or increased by a single dilution (Table 6).

**TABLE 5:** MICs of azole antifungals (μg/mL) and reference antibiotics against *C. difficile* after exposure to simulated gastric fluid

Drug	*C. difficile* strain					
	ATCC BAA-1870			ATCC 630		
	0 h	4 h	24 h	0 h	4 h	24 h
Miconazole	1	1	1	1	1	1
Econazole	1	2	2	1	2	2
Tioconazole	1	4	4	2	4	4
Vancomycin	1	1	1	1	0.5	0.5
Fidaxomicin	≤0.008	0.015	0.015	0.015	0.03	0.03

**TABLE 6:** MICs of azole antifungals (μg/mL) and reference antibiotics against *C. difficile* in the presence of simulated intestinal fluid

Drug	*C. difficile* strain					
	ATCC BAA-1870			ATCC 630		
	0 h	4 h	24 h	0 h	4 h	24 h
Miconazole	1	0.5	1	1	0.5	1
Econazole	1	1	2	1	0.5	2
Tioconazole	1	1	2	2	2	2
Vancomycin	1	1	2	1	0.5	0.5
Fidaxomicin	≤0.008	≤0.008	0.015	0.015	0.015	≤0.008

### Mechanistic investigations

Rapid bactericidal activity is often correlated with disruption or permeabilization of the bacterial cell membrane. To determine whether the killing of *C. difficile* was due to permeabilization of the bacterial cell membrane and leakage of cytosolic contents, we examined the impact of azole antifungals on *C. difficile* cell membrane integrity via cell permeability assays (propidium iodide uptake, protein leakage, and ATP leakage).

### Propidium iodide uptake assay

**Fig 3:**
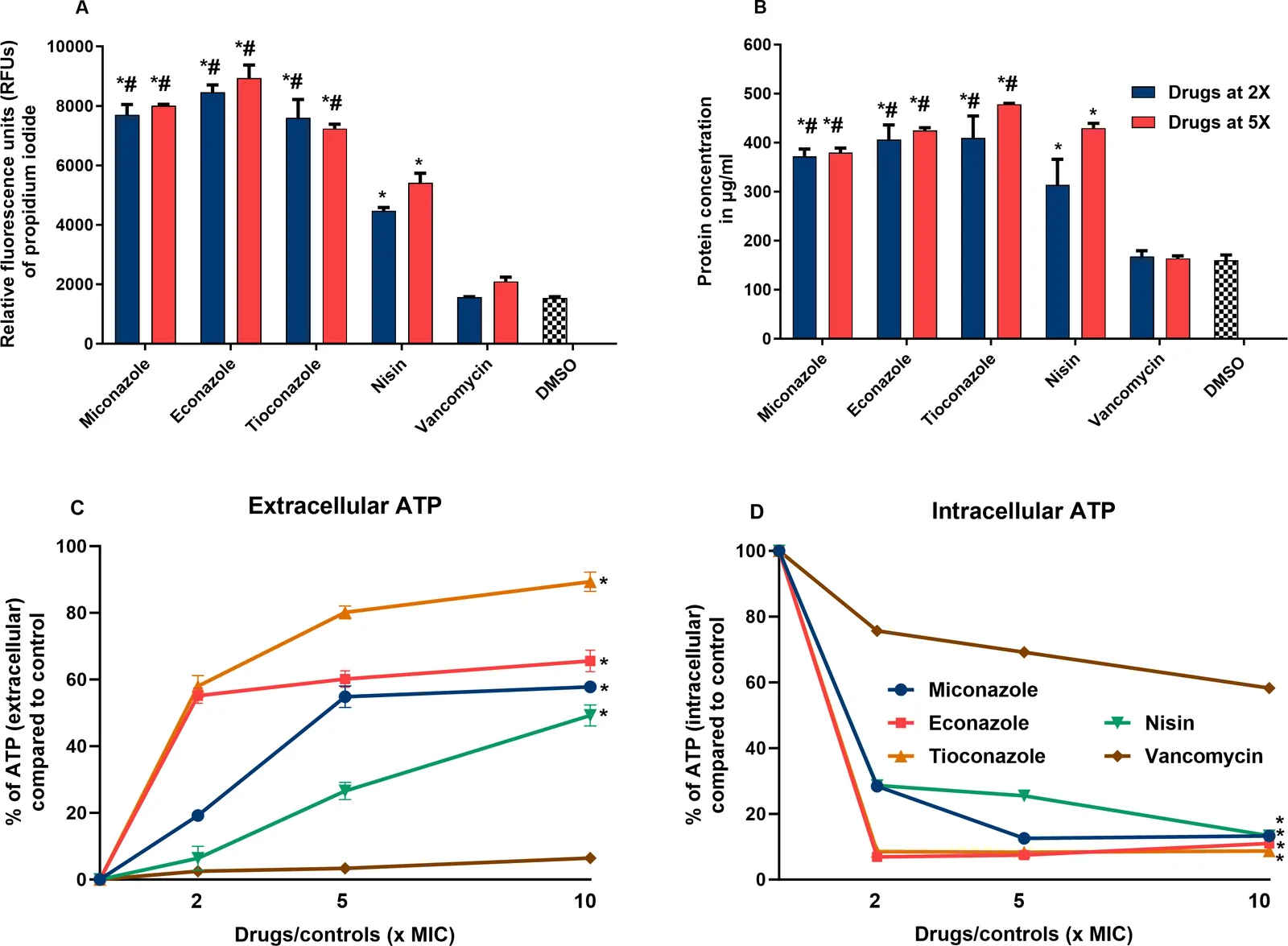
Mechanistic studies of the anti-*C. difficile* activity of azoles. (**A**) Effects of miconazole, econazole, and tioconazole (2× and 5× MIC) on the permeability of the bacterial cell membrane of *C. difficile* ATCC 43255, as determined via a PI uptake assay. The data are presented as the relative fluorescence units of PI in cells after treatment with different agents. A higher RFU value indicates a greater degree of cell membrane permeability. (**B**) Effects of miconazole, econazole, and tioconazole (2× and 5× MIC) on protein leakage from *C. difficile* ATCC 43255. The bacterial cells were subjected to azole drugs and the concentration of extracellular protein (ng/mL) was calculated. The data from the PI uptake and protein leakage assays were analyzed via two-way ANOVA followed by Dunnett’s test for multiple comparisons (*P <* 0.0001). Asterisks indicate a statistically significant difference between treatment with azoles or nisin and treatment with DMSO (negative control). Pounds (#) denote a statistically significant difference between treatment with azoles and nisin. (**C and D**) ATP leakage assay of azoles. *C. difficile* ATCC 43255 was exposed to azoles or nisin (2×, 5×, and 10× MIC), and the relative percentage of ATP, relative to the control (DMSO) either extracellularly (**C**) or intracellularly (**D**), was calculated and plotted.

The PI uptake assay demonstrated a concentration-dependent increase in PI fluorescence activity in response to azole treatment (Fig. 3A). Econazole displayed the highest fluorescence activity, with relative fluorescence units (RFUs) of ~8,000–9,000. Miconazole and tioconazole also presented high RFU values (~7,200–8,000). Notably, the PI fluorescence induced by the tested azoles surpassed the fluorescence elicited by nisin, the positive control for membrane permeabilization (61, 77, 78). The RFU of PI was ~4,400–5,400 for nisin-treated cells, whereas it was ~1,500 for the untreated control cells (DMSO). These results indicate that the azoles exert their antibacterial activity through permeabilization of the bacterial cell membrane. While vancomycin was included as a negative control in the assay, as expected, it showed similar activity to that of DMSO.

### Protein leakage assay

A bicinchoninic acid assay was used to determine the degree of intracellular protein leakage resulting from treatment with azoles (Fig. 3B). Nisin, a control antibiotic for increasing cell permeability, caused leakage of intracellular protein (between 300 and 430 ng/mL). Compared with the negative control, DMSO (160 ng/mL), and vancomycin (167 ng/mL), azole significantly increased protein leakage extracellularly. The fact that miconazole caused protein leakage (~370–380 ng/mL), whereas econazole and tioconazole caused more protein leakage (400–480 ng/mL), suggests that azoles target *C. difficile* cells by disrupting the permeability of the cell membrane.

### ATP leakage assay

The effects of miconazole, econazole, and tioconazole on the ATP leakage from a logarithmic-phase culture of *C. difficile* are shown in Fig. 3C and D. Compared with the untreated control (DMSO), nisin caused significant leakage of ATP, as indicated by both the intracellular (13%–30%) and the extracellular (5%–50%) levels of ATP. Exposure to the tested azoles at all concentrations tested (2×, 5×, and 10× MIC) resulted in a significant decrease in the level of intracellular ATP and a significant increase in the level of extracellular ATP after 3 h of exposure. Tioconazole resulted in increased levels of extracellular ATP (60%–90%) at the three tested concentrations. Econazole and miconazole generated lower levels of extracellular ATP (~55%–65%) and (20%–55%), respectively, at the three tested concentrations. Tioconazole and econazole resulted in similar levels of intracellular ATP (~10%–15%), whereas miconazole resulted in intracellular ATP levels ranging from ~15% to 30%.

### Imaging of *C. difficile* by scanning electron microscopy

**Fig 4:**
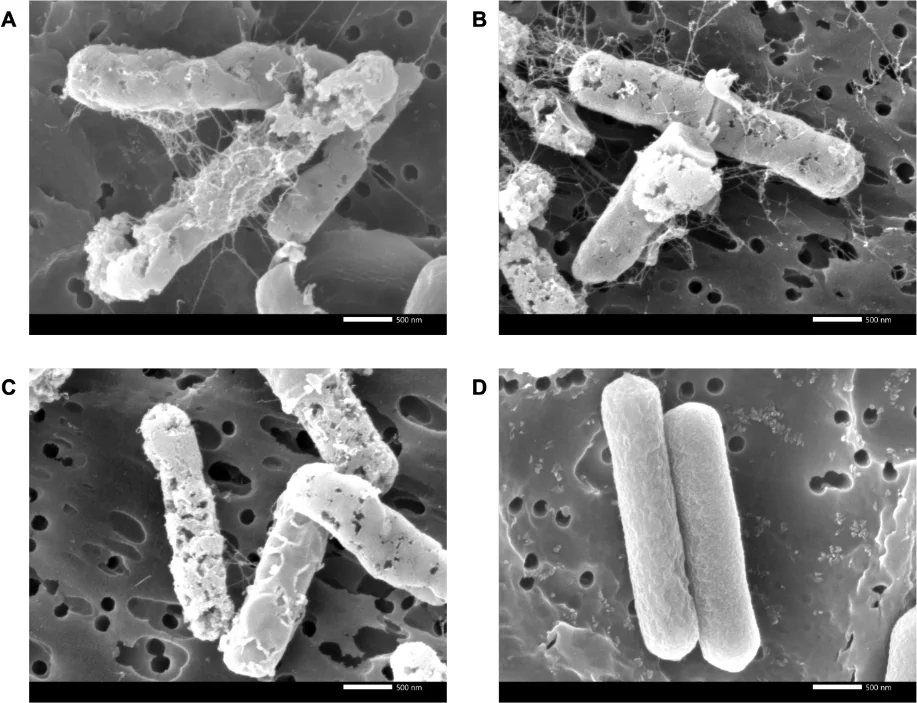
Scanning electron microscopy analysis of *C. difficile* following azole treatment. Scanning electron micrographs of logarithmic-phase *C. difficile* ATCC 43255 vegetative cells following exposure to azole compounds at 5× MIC: (**A**) miconazole, (**B**) econazole, and (**C**) tioconazole. Treated cells exhibit pronounced morphological alterations, including membrane distortion, surface irregularities, and cellular collapse. (**D**) Untreated control cells displaying intact membranes and preserved cellular morphology. Images shown in this figure are at 30,000× magnification. The corresponding full-field images at 2,500× and 10,000× magnifications are provided in Fig. S2 and S3.

The impact of azole treatment on the cellular morphology and membrane integrity of *C. difficile* was examined using SEM. Vegetative cells treated with azoles (miconazole, econazole, and tioconazole) at 5× MIC exhibited pronounced morphological abnormalities, including surface irregularities, membrane distortion, and cellular collapse. These structural alterations are indicative of severe membrane damage and compromised cellular integrity (Fig. 4A through C). In contrast, untreated control cells maintained a smooth cell surface and preserved rod-shaped morphology, consistent with intact membrane structure (Fig. 4D). These observations suggest that azole treatment disrupts the cellular envelope of *C. difficile*, contributing to the bactericidal activity of these compounds.

### Effect of cholesterol supplementation on the anti-*C. difficile* activity of the azole drugs

Previous studies have demonstrated that cholesterol incorporation enhances the ability of *Helicobacter pylori* to resist antibiotics and membrane-disrupting agents such as antimicrobial peptides and colistin (67). Given that intestinal pathogens routinely encounter cholesterol in the gut in the form of host membrane cholesterol, gastric mucus cholesterol, or dietary cholesterol, we sought to investigate whether exogenous cholesterol could alter the susceptibility of *C. difficile* to azole-mediated killing.

**Fig 5:**
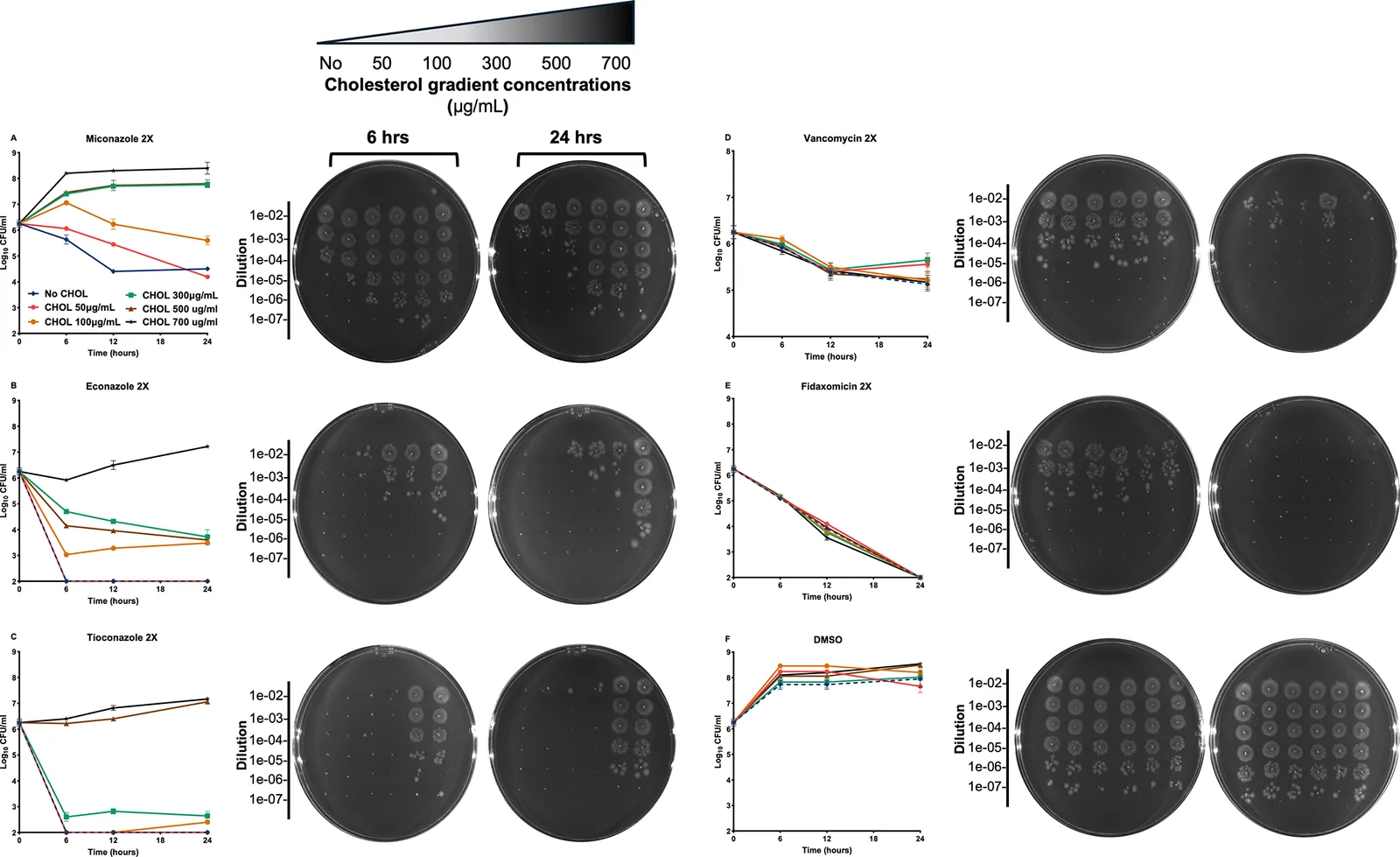
Exogenous cholesterol antagonizes the anti-*C. difficile* activity of membrane-disrupting azole drugs in a time-dependent manner. Time-kill curves showing the survival of *C. difficile* exposed to drugs at 2× MIC in the absence and presence of increasing concentrations of cholesterol (50, 100, 300, 500, and 700 µg/mL), including miconazole (**A**), econazole (**B**), tioconazole (**C**), vancomycin (**D**), fidaxomicin (**E**), and DMSO (**F**). Cultures were sampled at 0, 6, 12, and 24 h, serially diluted, and plated on BHIS agar to determine CFU/mL. The data represent the means ± SDs from three independent experiments. CHO, cholesterol; VAN, vancomycin; and FDX, fidaxomicin.

Supplementation with exogenous cholesterol had a dose-dependent antagonistic effect on the bactericidal activity of the tested azoles. Notably, increasing concentrations of cholesterol progressively diminished the anti-*C. difficile* efficacy of the azoles. For miconazole, cholesterol at 100 and 300 μg/mL resulted in modest antagonism, while concentrations of 500, 600, and 700 μg/mL completely abrogated its activity (Fig. 5A). Similarly, the activity of econazole was significantly reduced, with ~2-log CFU increases observed at 100–300 μg/mL cholesterol and complete loss of activity at 700 μg/mL (Fig. 5B). Tioconazole also exhibited a dose-dependent response, with partial antagonism at 300 μg/mL and complete loss of efficacy at 500 and 700 μg/mL (Fig. 5C).

In contrast, the efficacy of vancomycin (a cell wall synthesis inhibitor) and fidaxomicin (an RNA polymerase inhibitor) remained unaffected by cholesterol supplementation (Fig. 5D and E, respectively), indicating that the cholesterol effect is correlated with agents disrupting cytoplasmic membrane integrity only. Importantly, cholesterol alone had no inhibitory effect on *C. difficile* growth at concentrations up to 700 μg/mL (Fig. 5F).

### *In vivo* efficacy of azoles in the *C. elegans* infection model

Finally, the *C. elegans* infection model was used as a whole-animal system to evaluate the *in vivo* efficacy of azoles and to validate their promising *in vitro* activity. Untreated infected worms showed progressive mortality, reaching 73.3% by day 9.

**Fig 6:**
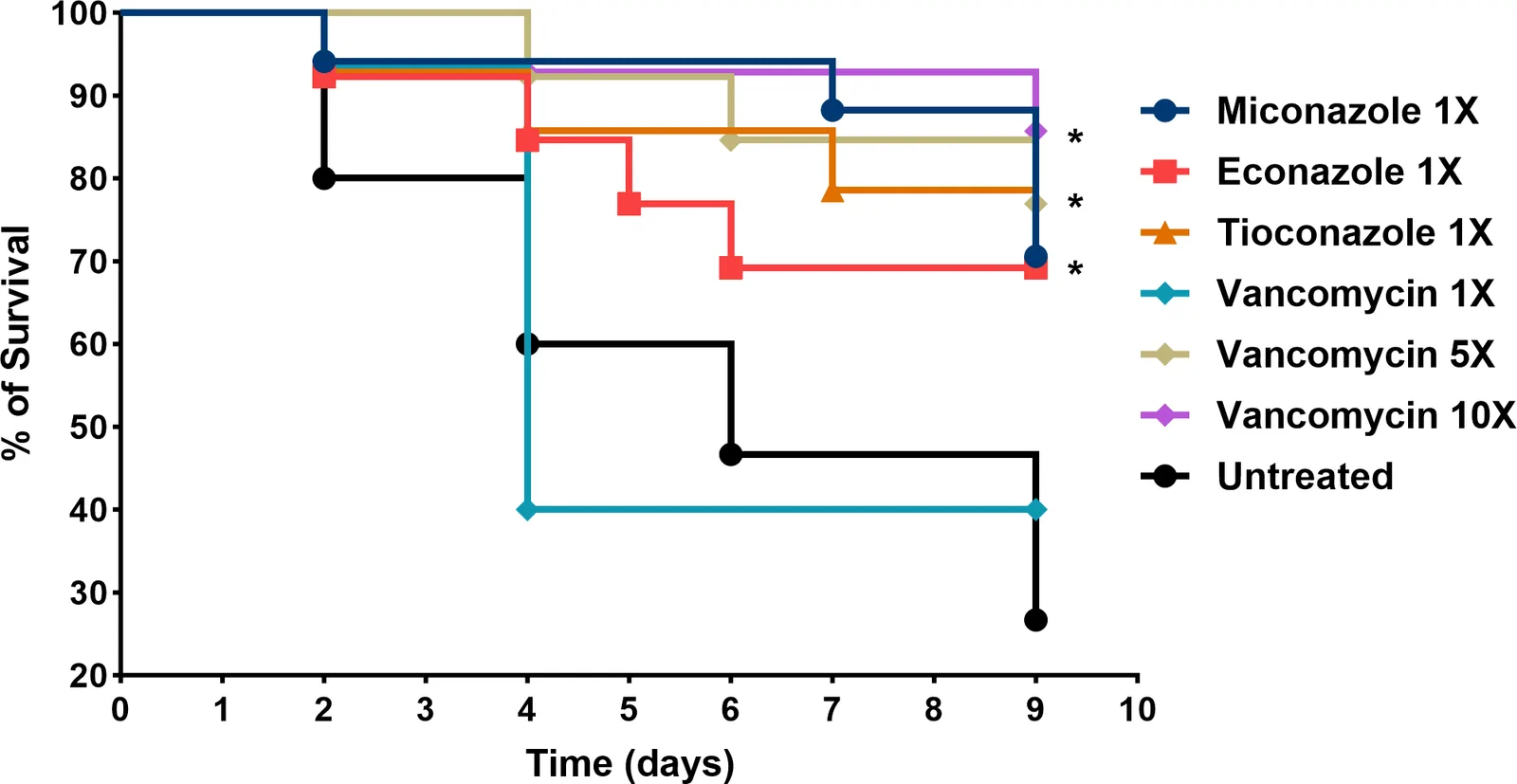
*In vivo* efficacy of azoles in a *C. elegans* infection model. *C. elegans* worms were infected with *C. difficile* ATCC 43255 and treated with miconazole, econazole, and tioconazole (at 1× MIC) and vancomycin (at 1×, 5×, and 10× MIC). Kaplan–Meier survival curves were analyzed via a log-rank (Mantel–Cox) test. Asterisks (*) denote a statistically significant difference between worms treated with miconazole, econazole, tioconazole, or vancomycin and untreated worms.

Treatment with miconazole, econazole, and tioconazole at 1× MIC significantly reduced mortality compared with untreated controls, resulting in survival rates of 70.5%, 69.2%, and 71.4%, respectively, after 9 days of treatment. In contrast, vancomycin at 1× MIC did not confer protection and showed mortality comparable to the untreated group. Higher concentrations of vancomycin were required to achieve significant protection, with 76.9% and 85.7% survival observed at 5× and 10× MIC, respectively. Notably, azoles at 1× MIC demonstrated protective efficacy comparable to high-dose vancomycin (10× MIC) in this model (Fig. 6).

These findings support the *in vivo* activity of the tested azoles in a whole-organism infection system and provide proof-of-concept evidence for further validation in mammalian CDI models.

## DISCUSSION

CDI is a serious and common cause of healthcare-associated diarrhea and is often associated with antibiotic use. Although vancomycin and fidaxomicin are currently approved for treatment, both have reports of treatment failure and CDI recurrence (28, 79). These challenges highlight the unmet need for new, effective, and affordable treatment options. Drug repurposing provides a faster and less expensive way to identify such alternatives. By adopting a drug repurposing strategy, we identified three azoles, miconazole, econazole, and tioconazole, as potent inhibitors of *C. difficile* (46). Owing to their well-defined safety profiles and FDA approval, these azoles represent viable anti-*C. difficile* candidates with favorable translational properties (80, 81). Importantly, these compounds exhibit limited gastrointestinal absorption; for example, miconazole demonstrates low oral bioavailability (25%–30%), which may allow preferential accumulation at the site of infection within the intestinal lumen (82).

Recent evidence indicates that fungal communities within the gastrointestinal tract (the gut mycobiome) play an important role in the pathophysiology of CDI, influencing host immune responses, microbial community structure, and disease severity in both humans and animal models (83, 84). Fungal overgrowth, particularly of *Candida* species, has been associated with increased susceptibility to CDI and heightened inflammatory responses (85, 86). In this context, azole antifungals were of particular interest not only for their antibacterial activity against *C. difficile* but also for their potential to modulate pathogenic fungal populations within the gut. The selection of azoles, therefore, reflects a strategic approach aimed at exploiting their dual antibacterial and antifungal activities, which may provide broader ecological control within the dysbiotic gut environment that is characteristic of CDI. Moreover, their limited systemic absorption, consistent with their established use in topical and mucocutaneous infections, supports their suitability for localized gastrointestinal activity while minimizing systemic exposure (87, 88).

Here in this study, we assessed the activity of the antifungal azoles (miconazole, econazole, and tioconazole) against a panel of *C. difficile* strains, including clinically toxigenic isolates. The azoles inhibited the growth of the strains tested at concentrations ranging from 1 to 4 μg/mL. Remarkably, the MICs of miconazole were comparable to those of vancomycin against the isolates tested, with an MIC_50_ of 1 μg/mL. Given that bactericidal agents are generally associated with improved clinical outcomes compared with bacteriostatic drugs (89), we performed a time-kill kinetics experiment to examine the bactericidal activity of the azoles against *C. difficile* inoculum. The tested azoles outperformed vancomycin and fidaxomicin in their ability to reduce the *C. difficile* burden, completely eradicating it within 2 h. Vancomycin resulted in a slow reduction in the *C. difficile* count, and fidaxomicin resulted in a gradual decrease in the count (2 log_10_ CFU/mL) after 12 h, which is consistent with earlier reports (90). This accelerated killing is likely attributable to their membrane-disruptive mechanism of action. Unlike vancomycin, which inhibits peptidoglycan synthesis (91), and fidaxomicin, which blocks RNA polymerase activity (92), azoles compromise cytoplasmic membrane integrity, leading to rapid leakage of intracellular contents and energetic collapse. Membrane-active agents typically induce faster bacterial death than inhibitors of macromolecular synthesis, consistent with the observed killing kinetics (93, 94). Rapid bactericidal activity is desirable for anti-*C. difficile* agents to control the progression of disease and significantly contribute to reducing the development of bacterial resistance to antibiotics (95).

Since the PAE of new antimicrobials is an important step that helps in determining the frequency of dosing, we determined the PAE of miconazole, econazole, and tioconazole after brief exposure to bacteria. Interestingly, the drugs produced prolonged PAEs ranging from 6 to ≥12 h, surpassing the drugs of choice, vancomycin and fidaxomicin, which exhibited PAE ranges of 2–6 h, similar to previous reports (96). Notably, extended PAEs are highly desirable, as they can reduce the dosing frequency, improve patient compliance, minimize toxicity, and lower treatment costs (97). On the other hand, the bacterial inoculum effect is a critical consideration in the development of effective anti-*C. difficile* drugs because *C. difficile* commonly colonizes the cecal and fecal contents of *C. difficile*-infected mice at approximately 10⁶–10⁷ CFU/g (98). Despite this, a lower bacterial inoculum (~10^5^ CFU/mL) is used in standard antimicrobial susceptibility assays. As the bacterial inoculum increased from 10^5^ to 10^7^ CFU/mL, the azole MICs remained equal to or onefold greater than the corresponding MICs of the standard inoculum, indicating that the anti-*C. difficile* activity of azoles was not affected by increasing the inoculum size. This contrasts with vancomycin, which exhibited a twofold increase in MICs at higher inoculum, whereas fidaxomicin maintained stable activity, with MIC values equivalent to or one dilution higher, aligning with previous reports (58).

Thus, we were curious to explore whether azole drugs impact the normal intestinal flora. A major drawback of the currently approved therapeutics for the treatment of CDI is their disruption of commensal bacteria, which contributes to dysbiosis, recurrence, and treatment failure. Ideally, anti-CDI agents should selectively target *C. difficile* while sparing beneficial microbes (99). Notably, miconazole, econazole, and tioconazole showed minimal to no activity against representative strains of *Lactobacillus* and *Bifidobacterium*, which are known to protect against CDI recurrence (100–102). While this assessment was limited to selected commensal species, these findings suggest a more selective antibacterial profile compared with vancomycin and fidaxomicin, which exhibited potent activity against some of the tested strains, consistent with previous reports (46, 103, 104).

Additionally, the stability of a drug under gastric and intestinal conditions provides insight into whether the orally administered anti-*C. difficile* drug is susceptible to degradation by GIT fluids before absorption occurs (105). To investigate the effects of gastric and intestinal conditions on anti-*C. difficile* activity of the azoles, their MICs against *C. difficile* were determined under mildly acidic to basic pH conditions, and their activity after exposure to simulated gastric and intestinal fluids (SGF and SIF) for up to 24 h was assessed. Notably, the MICs of miconazole, econazole, and tioconazole remained unchanged or showed only onefold variation under mildly acidic to basic pH conditions, unlike vancomycin and fidaxomicin, which presented increased MICs at higher pH values, which is consistent with previous studies (55). These results indicate that the tested azoles were advantageously capable of withstanding the harsh conditions of the GIT and that their activity was not affected by the pH conditions or enzymes present in the gastric or intestinal milieu, supporting their potential for oral delivery.

Agents that display rapid bactericidal activity are often associated with compromised membrane integrity, leading to swift bacterial killing (106, 107). Given the essential role of membrane integrity in bacterial survival, the clostridial membrane represents an attractive target for CDI therapy (61). For example, surotomycin was previously reported as a potent anti-*C. difficile* agent disrupting cytoplasmic membrane function (108). The drug reached phase III clinical trials; however, it failed to meet the criteria for noninferiority versus vancomycin (109). Thus, we evaluated whether the azoles exert their anti-*C. difficile* effects by disrupting *C. difficile* cytoplasmic membrane integrity. First, the results from the PI cell permeability assay revealed that the tested azoles induced concentration-dependent *C. difficile* cell membrane permeabilization. These results are consistent with their rapid bactericidal activity in the time-kill assay, where the tested azoles completely eradicated the high burden of *C. difficile* within 2 h. Nisin treatment induced high PI fluorescence compared with that of the untreated cells, which agrees with previous reports (60, 61). Vancomycin was included in the assay as a negative control since it does not disrupt the bacterial cell membrane and inhibits cell wall synthesis (110). As expected, the activity of vancomycin was similar to that of DMSO.

The exposure of bacterial cells to membrane-active agents is known to cause protein leakage and ATP depletion, which can arise from the uncoupling of ATP biosynthesis or from cell leakage due to membrane permeabilization (111). Consequently, to confirm that azoles target the *C. difficile* cytoplasmic membrane, we next examined protein and ATP leakage in *C. difficile* after exposure to azoles. Treatment with miconazole, econazole, or tioconazole resulted in a concentration-dependent reduction in the intracellular ATP and protein levels. These effects were more pronounced than those observed with the membrane-disrupting agent nisin, which elicited significant ATP and protein leakage, which is consistent with previous findings (60, 108). These results confirm that the disruption of the *C. difficile* membrane is a potential mechanism of action of azoles. Importantly, scanning electron microscopy further corroborated these findings by revealing substantial ultrastructural damage in azole-treated cells, including membrane distortion, surface irregularities, and cellular collapse. Together, the biochemical and ultrastructural evidence strongly supports membrane disruption as a primary mechanism underlying the anti*-C. difficile* activity of azoles.

Our cholesterol supplementation experiments in *C. difficile* are particularly noteworthy because, unlike *H. pylori*, nothing is currently known about the impact of cholesterol on this microorganism. Intestinal pathogens such as *H. pylori* and *C. difficile* encounter host cholesterol *in vivo* in various forms, including membrane-bound cholesterol, gastric mucus cholesterol, and dietary cholesterol. *H. pylori* is unique in that it incorporates cholesterol into its membrane and modifies it through α-glycosylation of the free hydroxyl group on the cholesterol steroid nucleus (67, 112–118). This incorporation and modification contribute to *H. pylori* pathogenicity in mice and are important for initial colonization in gerbils (113, 119). Cholesterol also enhances bacterial resistance to antibiotics and to membrane-disrupting agents such as antimicrobial peptides and colistin (67). In contrast, *C. difficile* is not known to synthesize sterols, and no direct evidence exists that it can incorporate cholesterol into its membrane. However, our data indicate that cholesterol supplementation enhances resistance to membrane-disrupting agents such as azoles and LL-37 (66–68). The mechanism underlying this effect remains unclear. Future studies are needed to determine the *in vivo* impact of cholesterol on *C. difficile* and to define how this process may influence its pathogenicity and susceptibility to different classes. Importantly, these findings raise the possibility that the host diet could directly influence *C. difficile* infection outcomes. If cholesterol availability alters bacterial resistance or virulence, dietary modulation of cholesterol intake might represent a novel clinical strategy to influence disease severity or therapeutic responsiveness in patients with *C. difficile* infection.

Finally, the *C. elegans* infection model was used as a whole animal model to evaluate the *in vivo* efficacy of azoles to validate the promising *in vitro* activity of the tested azoles. Miconazole, econazole, and tioconazole at low doses (1× MIC) significantly reduced the mortality rate of *C. difficile*-infected *C. elegans* worms compared with the untreated control, resulting in survival rates of 70.5%, 69.2%, and 71.4%, respectively, after 9 days of treatment. In contrast, vancomycin (1× MIC) did not protect the worms against CDI (100% mortality after 4 days). High concentrations of vancomycin (5× and 10× MIC) were needed to achieve significant protection against lethal CDI (76.9% and 85.7% survival, respectively).

In conclusion, these findings identify azole antifungals as promising candidates for repurposing against *C. difficile*, as they exhibit potent *in vitro* bactericidal activity, prolonged post-antibiotic effects, and limited disruption of the commensal gut microbiota. Their minimal impact on key gut commensals, together with protective efficacy in a *C. elegans* CDI model, provides important proof-of-concept support for their therapeutic potential. Importantly, the dual antibacterial and antifungal properties of azoles, combined with their limited gastrointestinal absorption and the prolonged post-antibiotic effect, suggest a strategic advantage for targeting both bacterial and fungal contributors to the dysbiotic gut environment associated with CDI.

It is worth mentioning that the present study has two limitations. First, it did not include pharmacokinetic analyses or evaluation in mammalian CDI models, which are essential for defining intestinal exposure, systemic absorption, and therapeutic efficacy in a clinically relevant context. Accordingly, future studies will focus on comprehensive PK profiling and validation in established murine CDI models to better assess translational potential. Notably, the membrane-targeting mechanism of these azoles distinguishes them from current CDI therapies and provides a strong foundation for structure-activity relationship-guided optimization aimed at enhancing gut confinement, potency, selectivity, and safety. Second, the potential impact of azole treatment on the gut mycobiome represents an important consideration and warrants further investigation in future *in vivo* studies incorporating multi-kingdom microbiome analyses. Collectively, these results support azole antifungals as valuable lead scaffolds for the development of next-generation therapeutics for CDI.

## SUPPLEMENTAL MATERIAL

Supplemental material spectrum.02900-25-s0001.docx Tables S1 and S2; Fig. S1 to S3.

ASM does not own the copyrights to Supplemental Material that may be linked to, or accessed through, an article. The authors have granted ASM a non-exclusive, world-wide license to publish the Supplemental Material files. Please contact the corresponding author directly for reuse.
