# Supplementary material for: Repurposing azole antifungals as rapid bactericidal, membrane-disruptive therapeutics against Clostridioides difficile
Source: Microbiol Spectr. 2026 May 18;14(7):e02900-25. doi: 10.1128/spectrum.02900-25 (PMC13340206; doi:10.1128/spectrum.02900-25)
Supplement: Supplemental material — Tables S1 and S2; Fig. S1 to S3. [file spectrum.02900-25-s0001.docx]

**Supplemental material**

**Repurposing Azole Antifungals as Rapid Bactericidal, Membrane-Disruptive Therapeutics Against *Clostridioides difficile***

Ahmed A. Abouelkhair^1,2^, Nader S. Abutaleb^1,2^, Brice J. Stolz^1,2^, Mohamed N. Seleem^1,2 *^

^1^ Department of Biomedical Sciences and Pathobiology, Virginia-Maryland College of Veterinary Medicine, Virginia Polytechnic Institute and State University, Blacksburg, VA 24061, USA.

^2^ Center for One Health Research, Virginia Polytechnic Institute and State University, Blacksburg, VA 24061, USA.

*Corresponding Author:

Mohamed N. Seleem

Department of Biomedical Sciences and Pathobiology

Virginia-Maryland College of Veterinary Medicine

Virginia Polytechnic Institute and State University

1410 Prices Fork Rd, Blacksburg, VA, 24061, USA

Phone: 540-231-2703

Email: seleem@vt.edu

**Table 1: Full description of the source and the characters of *C. difficile* strains used in this study.**

| **No.** | ***C.difficile* Strain** | **Alternate designation** | **Source** | **Characters** |
| --- | --- | --- | --- | --- |
| 1 | **NR- 49302** | *Clostridioides difficile* Isolate 20111075 | **BEI:** Stool of an elderly male patient with a healthcare-associated (HA) *C. difficile* infection in Minnesota, USA, in 2010. | *tcdA*, *tcdB* and *tcdC* of the PaLoc operon. This isolate is reported to be negative for the *C. difficile* binary toxin (CDT). |
| 2 | **NR- 49304** | *Clostridioides difficile* Isolate 20120956 | **BEI:** Stool of an older male patient with a healthcare-associated (HA) *C. difficile* infection in southern USA in 2011. | containing *tcdA*, *tcdB* and *tcdC* of the PaLoc operon. This isolate is reported to be negative for the *C. difficile* binary toxin (CDT). |
| 3 | **NR- 49306** | *Clostridioides difficile* Isolate 20110997 | **BEI:** Stool of an elderly male patient with a community-associated (CA) *C. difficile* infection in midwestern USA in 2011. | containing *tcdA*, *tcdB* and *tcdC* of the PaLoc operon. This isolate is reported to be negative for the *C. difficile* binary toxin (CDT). |
| 4 | **NR- 49307** | *Clostridioides difficile* Isolate 20120020 | **BEI:** Stool of an elderly female patient with a community-associated (CA) *C. difficile* infection in northeastern USA, in 2011. | containing *tcdA*, *tcdB* and *tcdC* of the PaLoc operon. This isolate is reported to be negative for the *C. difficile* binary toxin (CDT). |
| 5 | **NR- 49308** | *Clostridioides difficile* Isolate 20120166 | **BEI:** Stool of an elderly female patient with a community-associated (CA) *C. difficile* infection in Tennessee, USA, in 2011. | containing *tcdA*, *tcdB* and *tcdC* of the PaLoc operon. This isolate is reported to be negative for the *C. difficile* binary toxin (CDT). |
| 6 | **NR- 49310** | *Clostridioides difficile* Isolate 20110986 | **BEI:** Stool of a young adult male patient with a community-associated (CA) *C. difficile* infection in midwestern USA in 2011. | containing *tcdA*, *tcdB* and *tcdC* (with 39 base pair deletion) of the PaLoc operon as well as the *C. difficile* binary toxin (CDT). |
| 7 | **NR- 49313** | *Clostridioides difficile* Isolate 20110963 | **BEI:** Stool of an elderly female patient with a healthcare-associated (HA) *C. difficile* infection in Minnesota, USA, in 2011. | containing *tcdA*, *tcdB* and *tcdC* of the PaLoc operon. This isolate is reported to be negative for the *C. difficile* binary toxin (CDT). |
| 8 | **NR- 49314** | *Clostridioides difficile* Isolate 20121412 | **BEI:** Stool of a young female patient with a community-associated (CA) *C. difficile* infection in Georgia, USA, in 2011. | containing *tcdA*, *tcdB* and *tcdC* of the PaLoc operon. This isolate is reported to be negative for the *C. difficile* binary toxin (CDT). |
| 9 | **NR- 49318** | *Clostridioides difficile* Isolate 20110973 | **BEI:** Stool of a pediatric female patient with a community-associated (CA) *C. difficile* infection in midwestern USA in 2011. | containing *tcdA*, *tcdB* and *tcdC* of the PaLoc operon. This isolate is reported to be negative for the *C. difficile* binary toxin (CDT). |
| 10 | **NR- 49319** | *Clostridioides difficile* Isolate 20110992 | **BEI:** Stool of an elderly male patient with a community-associated (CA) *C. difficile* infection in midwestern USA in 2011. | containing *tcdA*, *tcdB* and *tcdC* of the PaLoc operon. This isolate is reported to be negative for the *C. difficile* binary toxin (CDT). |
| 11 | **CDC-1067** | *Clostridioides difficile* Isolate1067 | **CDC:** Human sample (Unknown) in 2016, USA (Ribotype -027). | It is positive for *tcdA, tcdB, cdtA, and cdtB* |
| 12 | **CDC-1072** | *Clostridioides difficile* Isolate1072 | **CDC:** Human sample (Unknown) in 2016, USA (Ribotype -027). | It is positive for *tcdA, tcdB, cdtA, and cdtB* |
| 13 | **CDC-1076** | *Clostridioides difficile* Isolate1076 | **CDC:** Human sample (Unknown) in 2016, USA (Ribotype -027). | It is positive for *tcdA, tcdB,* *cdtA,* and *cdtB* |
| 14 | **CDC-1077** | *Clostridioides difficile* Isolate1077 | **CDC:** Human sample (Unknown) in 2016, USA | It is positive for *tcdA, tcdB,* *cdtA,* and *cdtB* |
| 15 | **CDC-1078** | *Clostridioides difficile* Isolate1078 | **CDC:** Human sample (Unknown) in 2016, USA | It is positive for *tcdA, tcdB,* but negative with *cdtA, cdtB* |
| 16 | **CDC-1079** | *Clostridioides difficile* Isolate1079 | **CDC:** Human sample (Unknown) in 2016, USA | It is positive for *tcdA, tcdB,* but negative with *cdtA, cdtB* |
| 17 | **CDC-1082** | *Clostridioides difficile* Isolate1082 | **CDC:** Human sample (Unknown) in 2016, USA | It is positive for *tcdA, tcdB,* but negative with *cdtA, cdtB* |
| 18 | **CDC-1083** | *Clostridioides difficile* Isolate1083 | **CDC:** Human sample (Unknown) in 2016, USA | It is positive for *tcdA, tcdB,* *cdtA,* and *cdtB* |
| 19 | **CDC-1085** | *Clostridioides difficile* Isolate1085 | **CDC:** Human sample (Unknown) in 2016, USA | It is positive for *tcdA, tcdB,* but negative with *cdtA, cdtB* |
| 20 | **CDC-1086** | *Clostridioides difficile* Isolate 1086 | **CDC:** Human sample (Unknown) in 2016, USA | It is positive for *tcdA, tcdB,* but negative with *cdtA, cdtB* |
| 21 | **CDC-1087** | *Clostridioides difficile* Isolate1087 | **CDC:** Human sample (Unknown) in 2016, USA | It is positive for *tcdA, tcdB,* but negative with *cdtA, cdtB* |
| 22 | **CDC-1088** | *Clostridioides difficile* Isolate1088 | **CDC:** Human sample (Unknown) in 2016, USA | It is positive for *tcdA, tcdB,* but negative with *cdtA, cdtB* |
| 23 | **CDC-1089** | *Clostridioides difficile* Isolate1089 | **CDC:** Human sample (Unknown) in 2016, USA | It is positive for *tcdA, tcdB,* but negative with *cdtA, cdtB* |
| 24 | **CDC-1090** | *Clostridioides difficile* Isolate1090 | **CDC:** Human sample (Unknown) in 2016, USA | It is positive for *tcdA, tcdB,* but negative with *cdtA, cdtB* |
| 25 | **CDC-1092** | *Clostridioides difficile* Isolate1092 | **CDC:** Human sample (Unknown) in 2016, USA | It is positive for *tcdA, tcdB,* *cdtA,* and *cdtB* |
| 26 | **CDC-1093** | *Clostridioides difficile* Isolate1093 | **CDC:** Human sample (Unknown) in 2016, USA | It is positive for *tcdA, tcdB,* but negative with *cdtA, cdtB* |
| 27 | **CDC-1094** | *Clostridioides difficile* Isolate1094 | **CDC:** Human sample (Unknown) in 2016, USA | It is positive for *tcdA, tcdB,* *cdtA,* and *cdtB* |
| 28 | **CDC-1095** | *Clostridioides difficile* Isolate1095 | **CDC:** Human sample (Unknown) in 2016, USA (Ribotype -027). | It is positive for *tcdA, tcdB,* *cdtA,* and *cdtB* |
| 29 | **CDC-1096** | *Clostridioides difficile* Isolate1096 | **CDC:** Human sample (Unknown) in 2016, USA | It is positive for *tcdA, tcdB,* but negative with *cdtA, cdtB* |
| 30 | **ATCC 630** | *Clostridioides difficile* Isolate BAA-1382 | **ATCC:** Switzerland | It is positive for *tcdA, tcdB,* but negative with *cdtB* |
| 31 | **ATCC 43255** | *Clostridioides difficile* Isolate VPI 10463 | **ATCC:** Abdominal wound (Ribotype-087) | It is positive for *tcdA, tcdB,* but negative with *cdtB* |
| 32 | **ATCC BAA-1870** | *Clostridioides difficile* Isolate 4118 | **ATCC:** Clinical isolate (Ribotype-027) | It is positive for *tcdA, tcdB,* and *cdtB* |
| 33 | **ATCC 9689** | *Clostridioides difficile* Isolate 90556-M6S | **ATCC:** Clinical isolate (Ribotype-001) | It is positive for *tcdA, tcdB,* but negative with *cdtB* |

**CDC**; The Centers for Disease Control and Prevention.

**BEI Resources**; The Biodefense and Emerging Infections Research Resources Repository.

**ATCC**; The American Type Culture Collection.

**Table 2: Full description of the source and the characters of gut microbiota used in the study.**

| **No.** | **Common gut bacteria** | **Alternate designation** | **Source** |
| --- | --- | --- | --- |
| 1 | **HM-846 *Bifidobacterium longum*** | 1-6B | Feces of a six-year-old healthy human child in Russia. |
| 2 | **HM-1189 *Bifidobacterium angulatum*** | F16_22 | Human stool in Guelph, Ontario, Canada. |
| 3 | **HM-845 *Bifidobacterium longum*** | 44B | A one-year-old human patient. |
| 4 | **HM-847 *Bifidobacterium longum*** | 35B | A one-year-old human patient. |
| 5 | **ATCC 15700 *Bifidobacterium breve*** | S1 (Variant a) | Intestine |
| 6 | **HM-856 *Bifidobacterium breve*** | HPH0326 | Biopsy of ileo-anal pouch mucosa of a human subject in the United States. |
| 7 | **HM-633 *Bifidobacterium adolescentis*** | L2-32 | Fecal sample of a healthy two-year-old infant in Aberdeen, Scotland, United Kingdom. |
| 8 | **ATCC 53103 *Lacticaseibacillus rhamnosus*** |  | Faeces |
| 9 | **ATCC 19992 *Lactobacillus gasseri*** |  | Faeces |
| 10 | **ATCC 14869 *Levilactobacillus brevis*** | Bb14 | Faeces |
| 11 | **HM-228 *Lactobacillus iners.*** |  | Faeces |
| 12 | **ATCC 334 *Lactobacillus casei*** |  | Dairy products |


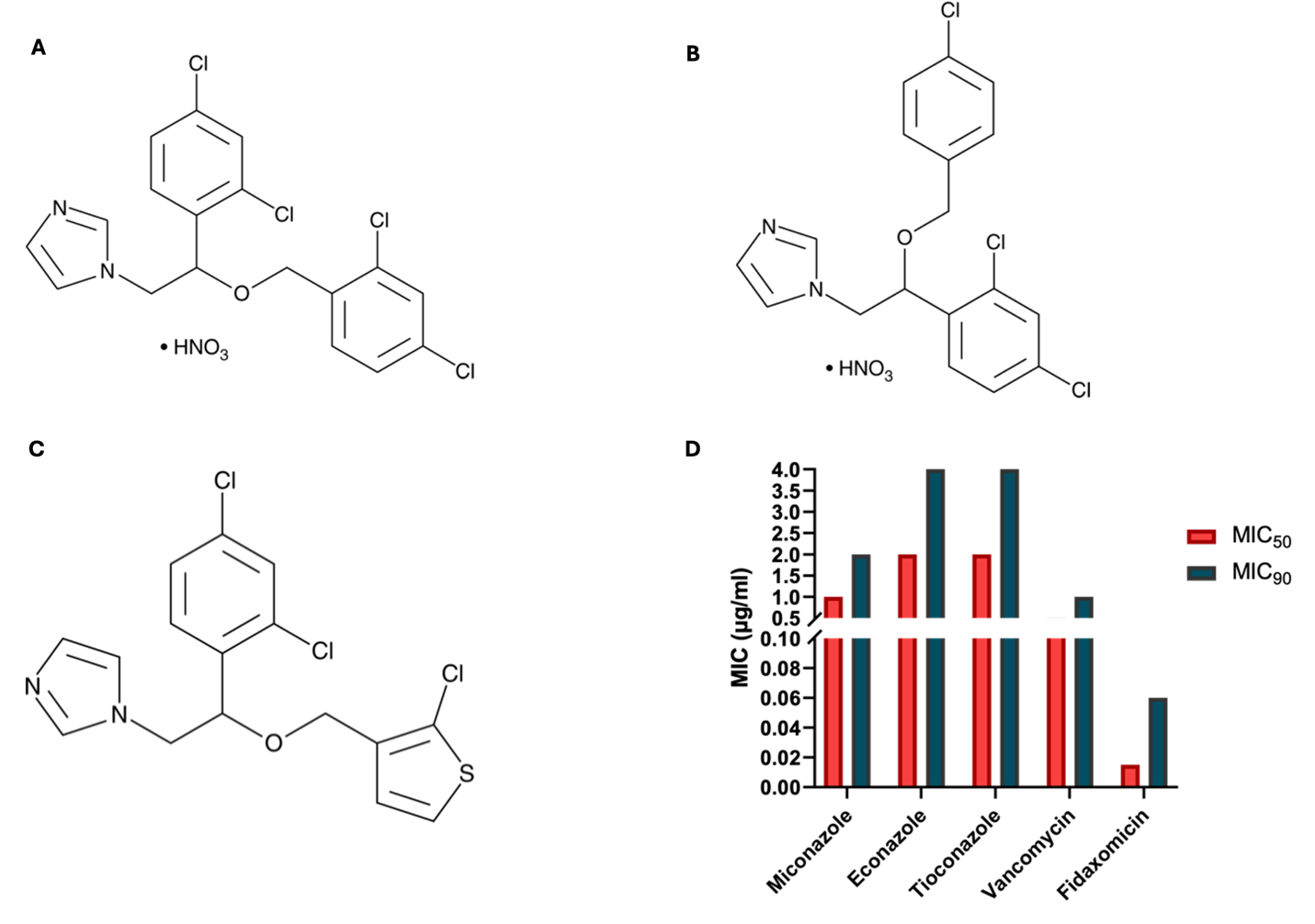


**Figure. S1.** **Azole antifungals have anti-*C. difficile* properties.** Azole’s chemical structures; (**A**) miconazole, (**B**) econazole, and (**C**) tioconazole. (**D**) Azoles' MIC_50_ and MIC_90_ values for 33 isolates (values in $\mu$g/ml).

**
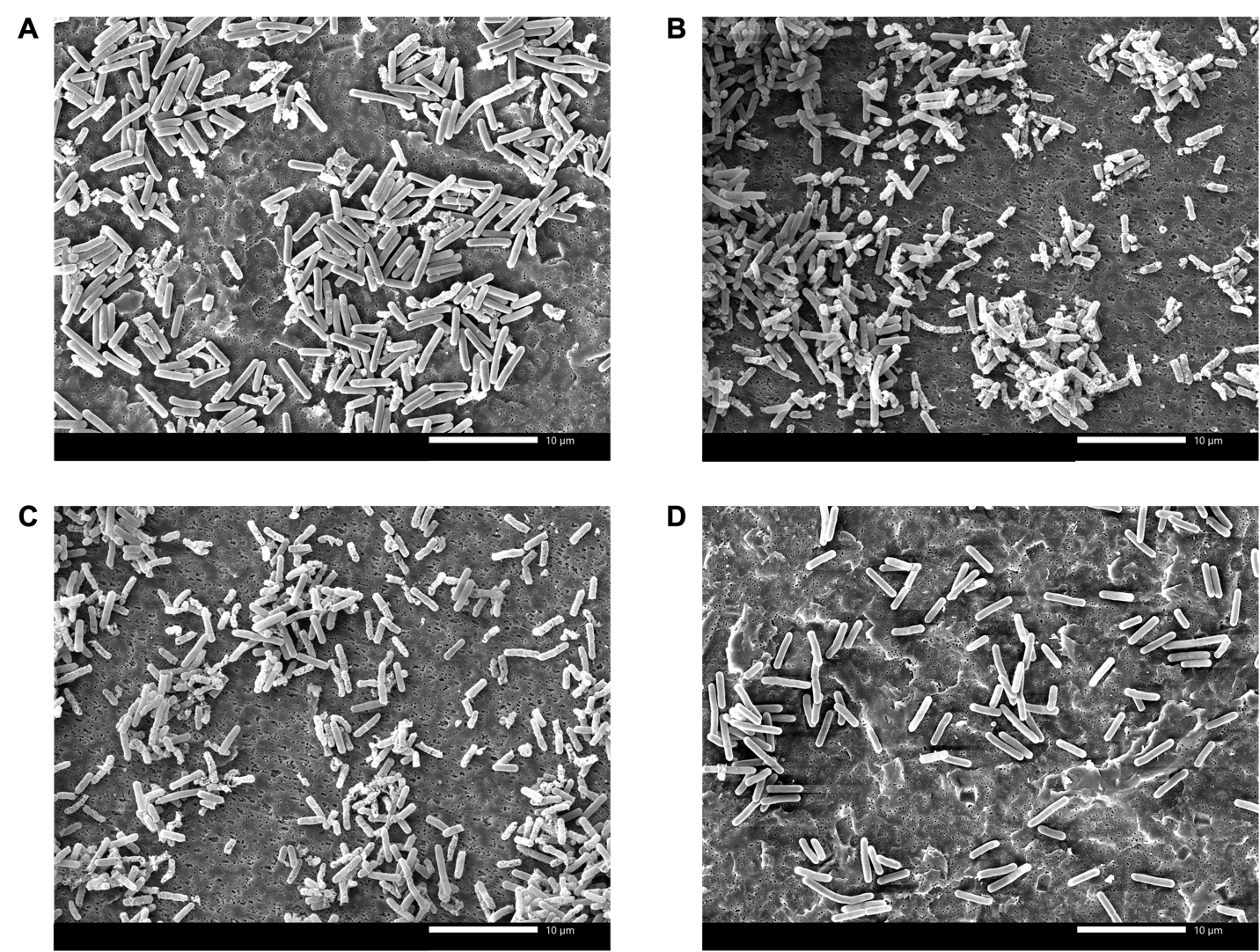
**

**Figure. S2.** **Scanning electron microscopy analysis of *C. difficile* following azole treatment.** Scanning electron micrographs of logarithmic-phase *C. difficile* ATCC 43255 vegetative cells following exposure to azole compounds at 5× MIC: (**A**) miconazole, (**B**) econazole, and (**C**) tioconazole. Treated cells exhibit pronounced morphological alterations, including membrane distortion, surface irregularities, and cellular collapse. (**D**) Untreated control cells displaying intact membranes and preserved cellular morphology. Images shown in this figure are at 2,5000× magnification.

**
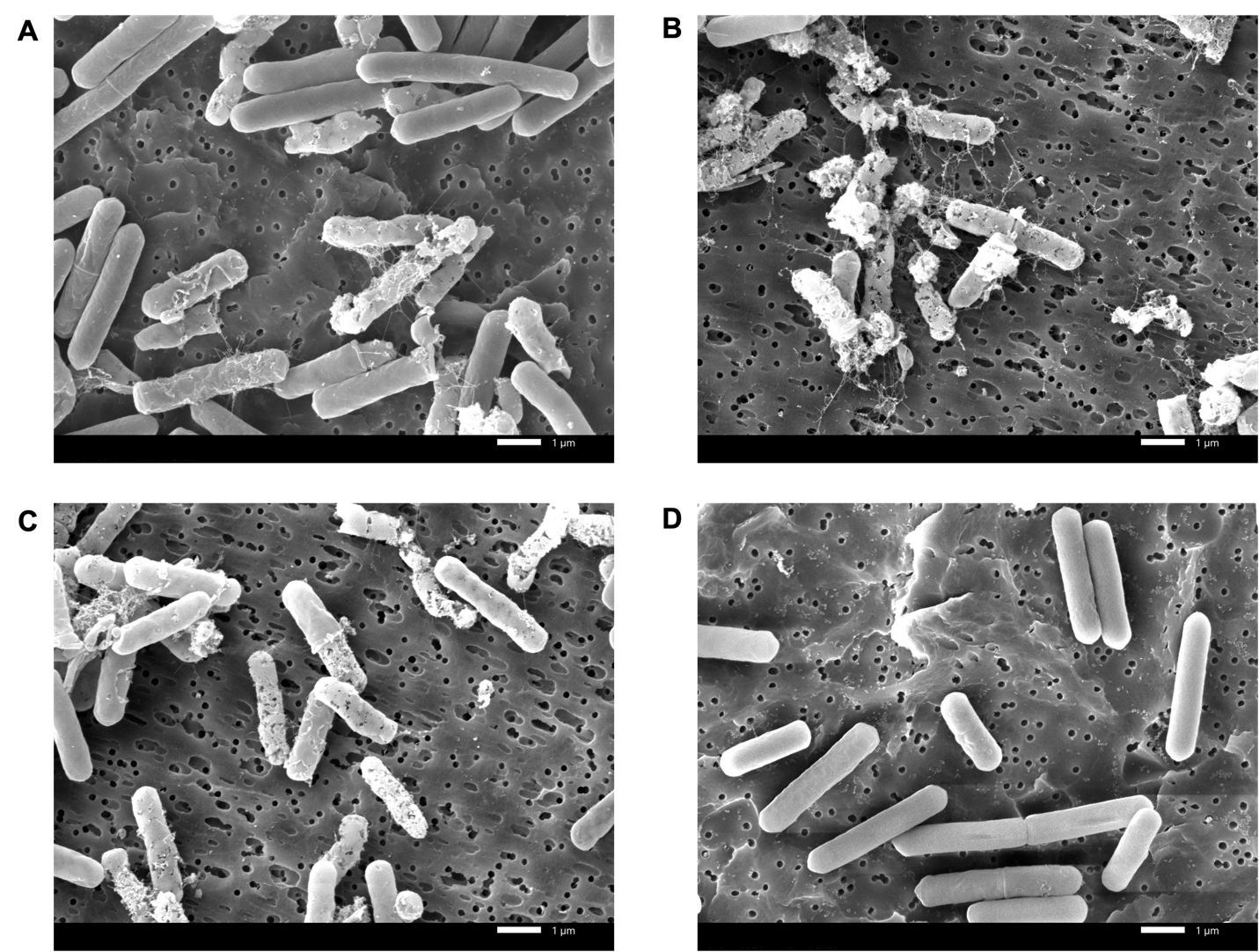
**

**Figure. S3.** **Scanning electron microscopy analysis of *C. difficile* following azole treatment.**Scanning electron micrographs of logarithmic-phase *C. difficile* ATCC 43255 vegetative cells following exposure to azole compounds at 5× MIC: (**A**) miconazole, (**B**) econazole, and (**C**) tioconazole. Treated cells exhibit pronounced morphological alterations, including membrane distortion, surface irregularities, and cellular collapse. (**D**) Untreated control cells displaying intact membranes and preserved cellular morphology. Images shown in this figure are at 10,000× magnification.
